# Factor structure of PTSD, and relation with gender in trauma survivors from India

**DOI:** 10.3402/ejpt.v5.25547

**Published:** 2014-11-17

**Authors:** Ruby Charak, Cherie Armour, Ask Elklit, Disket Angmo, Jon D. Elhai, Hans M. Koot

**Affiliations:** 1 Department of Developmental Psychology, VU University, Amsterdam, The Netherlands; 2 Department of Psychology, University of Jammu, Jammu, India; 3 School of Psychology, University of Ulster, Coleraine, UK; 4 The National Centre for Psychotraumatology, University of Southern Denmark, Odense, Denmark; 5 Government Eliezer Joldan Memorial College, Leh, India; 6 Department of Psychology, University of Toledo, Toledo, OH, USA; 7 Department of Psychiatry, University of Toledo, Toledo, OH, USA

**Keywords:** PTSD, factor structure, gender, Dysphoric Arousal model, MIMIC, natural disaster, displacement due to cross border conflict

## Abstract

**Background:**

The factor structure of posttraumatic stress disorder (PTSD) has been extensively studied in Western countries. Some studies have assessed its factor structure in Asia (China, Sri Lanka, and Malaysia), but few have directly assessed the factor structure of PTSD in an Indian adult sample. Furthermore, in a largely patriarchal society in India with strong gender roles, it becomes imperative to assess the association between the factors of PTSD and gender.

**Objective:**

The purpose of the present study was to assess the factor structure of PTSD in an Indian sample of trauma survivors based on prevailing models of PTSD defined in the DSM-IV-TR (APA, 2000), and to assess the relation between PTSD factors and gender.

**Method:**

The sample comprised of 313 participants (55.9% female) from Jammu and Kashmir, India, who had experienced a natural disaster (*N*=200) or displacement due to cross-border firing (*N*=113).

**Results:**

Three existing PTSD models—two four-factor models (Emotional Numbing and Dysphoria), and a five-factor model (Dysphoric Arousal)—were tested using Confirmatory Factor Analysis with addition of gender as a covariate. The three competing models had similar fit indices although the Dysphoric Arousal model fit significantly better than Emotional Numbing and Dysphoria models. Gender differences were found across the factors of Re-experiencing and Anxious arousal.

**Conclusions:**

Findings indicate that the Dysphoric Arousal model of PTSD was the best model; albeit the fit indices of all models were fairly similar. Compared to males, females scored higher on factors of Re-experiencing and Anxious arousal. Gender differences found across two factors of PTSD are discussed in light of the social milieu in India.

The release of the DSM-5 comes with a number of amendments to the nosology of posttraumatic stress disorder (PTSD), including the addition of three symptoms (negative expectation of oneself/world/others, distorted blame, and recklessness), a revision of existing symptoms, and a division of symptoms across four rather than the earlier three symptom groups (American Psychiatric Association [APA], 2013). This latter decision was influenced by the factor analytic support garnered by two alternative four-factor models of Emotional Numbing (King, Leskin, King, & Weathers, 1998), and Dysphoria (Simms, Watson, & Doebbeling, 2002). Notably, the DSM-5 version of the factor structure of PTSD is more similar to the Emotional Numbing model, which emerged as a result of the bifurcation of the DSM-IV-TR Avoidance/Numbing factor of PTSD (APA, 2000) into the factors of Avoidance, and Numbing, next to Re-experiencing, and Hyperarousal factors. More specifically, the four-factor model of PTSD put forth in the DSM-5 includes Re-experiencing, Avoidance, Negative alterations in mood and cognitions (NAMC; similar to the Numbing factor in the Emotional Numbing model; Forbes et al., 2011), and Alterations in arousal and reactivity (similar to the factor of Hyperarousal in the Emotional Numbing model) symptom clusters (Friedman, 2013). It is, however, pertinent to acknowledge recent research which has begun to build support for a five-factor model known as the Dysphoric Arousal model (Elhai et al., 2011). This model modifies the Emotional Numbing model by separating the Hyperarousal symptoms into two groupings termed Dysphoric Arousal and Anxious Arousal resulting in five distinct PTSD symptom groups. Using DSM-IV-TR based items, the current study will investigate which of the offered models of PTSD best represents the underlying dimensionality of PTSD in a sample of Indian trauma survivors. Given that the resultant structure of PTSD should be culturally generalizable in order to be valid, it is pertinent to assess existing models in such a sample since this type of research informs revisions to diagnostic criteria.

The Emotional Numbing model of PTSD (King et al., 1998) was first tested in treatment-seeking male veterans (*N*=524) which resulted in four correlated factors of Re-experiencing (B1–B5), Avoidance (C1–C2), Numbing (C3–C7), and Hyperarousal (D1–D5). The empirically substantiated distinction between the factors of Avoidance and Numbing is depicted by their differential relations with alternative psychopathological constructs (e.g., depression; Palmieri, Marshall, & Schell, 2007), and treatment outcomes (Asmundson, Stapleton, & Taylor, 2004). The model was thus superior to the tripartite model of PTSD in the DSM-IV-TR (APA, 2000), which was formulated primarily on the basis of expert consensus. Most recently, support for the model has been found in samples of military personnel (Mansfield, Williams, Hourani, & Babeu, 2010), medical patients (Naifeh, Elhai, Kashdan, & Grubaugh, 2008), refugees (Palmieri et al., 2007), and other trauma-affected populations (Elhai & Palmieri, 2011).

The Dysphoria model (Simms et al., 2002), also originally tested in a large sample of veterans (*N*=3,695), consists of four correlated factors, termed Re-experiencing (B1–B5), Avoidance (C1–C2), Dysphoria (C3–C7 and D1–D3), and Hyperarousal (D4–D5), and differs from the Emotional Numbing model only in the placement of three items (D1–D3). The Dysphoria factor extracts three items from Hyperarousal and combines them with the five items from the Avoidance/Numbing factor of the DSM-IV-TR PTSD model, creating a factor comprising eight items, representative of underlying general distress. Confirmatory factor analysis (CFA)-based studies have supported this model across a range of trauma populations such as military veterans (Pietrzak, Goldstein, Malley, Rivers, & Southwick, 2010), motor-vehicle accident victims (Elklit & Shevlin, 2007), and bereaved parents, survivors of rape, and refugees (Elklit, Armour, & Shevlin, 2009).

A meta-analysis of 40 studies favored the Dysphoria model over the Emotional Numbing model, but only marginally so (Yufik & Simms, 2010). Essentially, the difference between the two four-factor models lies in the items indicative of sleep disturbances, irritability, and difficulty in concentration (D1–D3). More recently, Elhai et al. (2011) stated that the changes in the Dysphoria model, that is, the extraction of three symptoms from Hyperarousal and their addition to the symptoms of Numbing factor, to form the Dysphoria factor, does not clarify which of the two modifications resulted in superior fit for the model. This point combined with an existing argument that items D1–D3 are conceptually different from the symptoms of Dysphoria and Hyperarousal (Shevlin, McBride, Armour, & Adamson, 2009; Watson, 2005), led Elhai et al. (2011) to propose that the three items (D1–D3) which differ in their placement in the two four-factor models represent a separate and unique PTSD factor. Therefore, they proposed a model comprising five separate factors termed Re-experiencing (B1–B5), Avoidance (C1–C2), Numbing (C3–C7), Dysphoric Arousal (D1–D3), and Anxious Arousal (D4–D5). Recent studies have also examined the relation between the five factors of the Dysphoric Arousal model of PTSD, and external measures of psychopathology (e.g., depression, anxiety, health-related quality of life; Tsai, Whealin, Scott, Harpaz-Rotem, & Pietrzak, 2012; Wang, Li, et al., 2011; Wang, Long, Li, & Armour, 2011). For example, a study on 323,903 US veterans assessing the factor structure of PTSD indicated that the Numbing factor of the Dysphoric Arousal model was most strongly associated with a diagnosis of depression and substance use disorder, and the Dysphoric Arousal factor was most strongly related to a diagnosis of anxiety disorder (Harpaz-Rotem, Tsai, Pietrzak, & Hoff, 2014).

Taken together, these studies provide support for the theoretical model proposed by Watson (2005) which separates symptoms involving restlessness and agitation (D1–D3) from more fear-based and physiological symptoms (D4–D5) that characterize PTSD. Furthermore, the items of sleep difficulties, irritability, and difficulties in concentration (D1–D3) are differentiated from the Numbing symptoms (C3–C7) as the former represents agitation and restlessness compared to a numbing of responsiveness. The factor analytic support for the Dysphoric Arousal model has grown substantially and has been report across victims from various countries, including China (Wang, Long, et al., 2011; Wang, Zhang, et al., 2011), Canada (Armour et al., 2012), the United States (Armour, Carragher, & Elhai, 2013; Harpaz-Rotem et al., 2014; Pietrzak et al., 2014; Pietrzak, Tsai, Harper-Rotem, Whealin, & Southwick, 2012), Australia (Armour, Carragher, et al., 2013), Sri Lanka (Semage et al., 2013), Denmark (Armour, O'Connor, Elklit, & Elhai, 2013), and Malaysia (Armour, Ghazali, & Elklit, 2013).

The developing support for the Dysphoric Arousal model (Elhai et al., 2011) and the release of DSM-5 which proposes the four-factor model of PTSD (APA, 2013) coincided, and hence it is likely that the Dysphoric Arousal model may not have gained sufficient momentum to be considered for DSM-5. Recently two six-factor models of PTSD based on DSM-5 PTSD symptoms, built on the Dysphoric Arousal model (the version based on DSM-5 PTSD symptoms) and add a sixth factor (see Liu et al., 2014; Tsai et al., in press). The newly proposed six-factor “Anhedonia model” retained the separation of Hyperarousal (as also in Dysphoric Arousal model), and separated the NAMC factor (of DSM-5 PTSD) into two constructs of “Negative alterations in cognitions and mood” and “Anhedonia” (Liu et al., 2014). This was conducted on a sample of Chinese earthquake survivors (*N*=1,196). On the other hand, the six-factor “Externalizing model” proposed by Tsai et al. (in press) assessed for PTSD factor structure in a nationally representative sample of US veterans (*N*=1,484). It found acceptable fit for a PTSD model which retained the separation of the Dysphoric Arousal and Anxious Arousal factors, and additionally created a new factor measuring “Externalizing behavior” (items: irritability or aggressive behavior and self-destruction or reckless behavior). Taken together, these recent studies indicate the ongoing debate regarding the factor structure of PTSD.

In the present study, we assessed for the factor structure of PTSD in an Indian sample of trauma victims based on DSM-IV-TR symptoms of PTSD as the assessment of the four-factor model described in the DSM-5, and the two six-factor models of PTSD were beyond the scope of this paper. In addition, we adhere to the call of addressing the need for more trauma-based research studies from low- to middle-income countries such as India (Fodor et al., 2014).

Some factor analytic studies have begun to look at characteristics (e.g., gender, age) that may account for differences in the factor structure of PTSD (Armour et al., 2011; Contractor et al., 2013). Gender is of particular interest in the present sample, considering it may have a specific role to play in a largely patriarchal Indian society. Numerous studies report that PTSD prevalence is two-fold in females compared to males (Tolin & Foa, 2006), despite evidence of a greater number of trauma exposures among males (Gavranidou & Rosener, 2003; Kessler, Sonnega, Bromet, Hughes, & Nelson, 1995). Several explanations have been forwarded for these sex-linked differences in PTSD. Factors of influence that have been mentioned are differences in cognitive appraisal, physiological vulnerability, socialization that supports active behavior and underreporting in males, and fearfulness and passive–avoidant behavior in females, and social factors such as the lack of social support and negative response that females face post-trauma (e.g., after sexual assault/rape; Gavranidou & Rosener, 2003; Norris, Perilla, Ibañez, & Murphy, 2001; Olff, Langeland, Draijer, & Gersons, 2007). However, there are also studies that find few differences in PTSD related to gender, and most have been conducted among military personnel (Brewin, Andrews, & Valentine, 2000; King, Street, Gradus, Vogt, & Resick, 2013). In addition, one prospective study on victims of motor vehicle collisions found no gender difference on prevalence or recovery from PTSD, across 1- and 4-month time periods (Freedman et al., 2002). Moreover, studies related to PTSD from India, often fail to provide evidence for the two-fold increase in PTSD among females when compared with males (Contractor et al., 2014; Kar et al., 2007). With such mixed results for gender differences in PTSD, we analyzed gender as a covariate in the present study.

Many Asian-sample studies indicate the presence of somatic symptoms as an expression of general distress in victims of traumatic stress (Kar et al., 2007; Terheggen, Stroebe, & Kleber, 2001). Indian culture, like many other Asian cultures, is collectivistic and fatalistic, and harbors interdependency while inhibiting self-identity (Sinha, 1984). For example, a study on victims of the Asian Tsunami from India highlighted that the community collectivized personal trauma, constructed meaning following the disaster using a fatalistic perspective, displayed mourning openly, and employed spiritual beliefs as coping mechanisms (Rajkumar, Premkumar, & Tharyan, 2008). Such behavior can lead to changes in the presentation of symptomatology consequent to the traumatic event, and may also result in variation in the underlying latent structure of PTSD. Given recent literature favoring the Dysphoric Arousal model in samples from Asian societies, including Malaysia (Armour, O'Connor, et al., 2013), Sri Lanka (Semage et al., 2013), and China (Wang, Long, et al., 2011; Wang, Zhang, et al., 2011), it is possible that the same underlying latent structure of PTSD will be evident in the present sample of trauma survivors from India. Further, the Indian society is largely patriarchal with females engaging in child-rearing and household activities, while the males are considered bread-winners and decision makers (Segal, 1999). Studies on PTSD prevalence from India suggest a higher rate in females compared to males (John, Russell, & Russell, 2007; Kumar et al., 2007) consistent with studies from the west. However, certain expressions of distress (e.g., crying, ruminating) or reporting feelings of distress or not being in control of a situation may not be considered acceptable for males in the Indian society. In line with this, a cross-cultural PTSD study assessing the role of gender and culture among disaster victims from Mexico (with traditional gender roles), and among African American families (non-traditional gender roles) indicated that the Mexican culture increased and the African American culture attenuated, differences in posttraumatic stress of males and females. Females scored consistently higher on the factor Arousal (as per DSM-IV-TR) than males across both the cultures (Norris et al., 2001). Such findings substantiate the influence of culturally sanctioned gender-roles on certain PTSD symptoms.

Against this background, the present study aimed to assess the factor structure of PTSD in a sample of trauma survivors from Jammu and Kashmir, India. Based on prior research from Asia, we hypothesized that the Dysphoric Arousal model would provide a superior fit when compared with models of Emotional Numbing and Dysphoria in the Indian sample of trauma survivors. Second, we aimed to assess the relation between gender and the factors of PTSD, obtained from CFA using a multiple indicator multiple causes (MIMIC) structural equation model. Based on previous literature, we hypothesized that the factors of PTSD would be associated with gender, with females scoring higher than the males on all factors. To the best of our knowledge, this is the first study to directly assess the factor structure of PTSD in an Indian sample of adult trauma victims, while assessing for gender difference across the factors of PTSD.

## Methods

### Participants

The present study included 313 participants (*M*_*age*_ *=*34.9, *SD*=12.3; 55.9% female) from two samples of trauma survivors from Jammu and Kashmir, which is the northern state of India. Sample 1 included 200 participants affected by flash floods as a result of cloudburst/heavy rainfall over the Leh (Ladakh) region in August 2010. Participants ranged in age from 19 to 76 years (*M*_*age*_ *=*34.75, *SD=*13.72), and over half of the sample was female (57.5%). Participants report a loss of property (48%), witnessing the floods (41.5%), and losing a loved one (8%) or losing both a loved one and property (2.5%) as a direct result of the disaster. All assessments were carried out between February and June 2011. Sample 2 included 113 participants living near the Line of Control (LoC), a de facto border between India and Pakistan, located in the Doyian village in the Akhnoor region. Participants were between the ages of 20 and 55 years (*M*_*age*_ *=*35.08, *SD=*9.27) and over half of the sample was female (53.1%). In the second sample, participants had been exposed to shelling and mortar firing during the initial phase of the conflict before being relocated to safer grounds. Assessment was carried out between January and March 2010, after their return to Doyian in 2004.

### Procedures

Data were collected individually in the participant's residence with the assistance of two graduate students (LS and MM) after explaining the purpose of the study. In sample 1, 13.5% of participants needed assistance in filling out the questionnaire as their comprehension of English (and Hindi, the national language) was low. In such cases, the assessor (LS) verbally translated the measure to Ladakhi (the local language). In Sample 2, nearly all of the participants (91.2%) requested assistance in filling in the questionnaire. Verbal translations were provided by the assessor (MM) in a dialect of the Dogri language spoken in the region. Notably, the two languages employed were neither the national nor the official state language, and back translation was not carried out. Participants in the diagnosable range of PTSD were asked to visit the nearest Health Center, following a session on psycho-education by the assessor. In the absence of an ethical committee at the University of Jammu where the study was designed, the research design was approved by the Chairperson and a faculty member at the Department of Psychology, University of Jammu, and was conducted in accordance with the Helsinki Declaration.

### Measure

Brief demographic details like age and gender (female=0, male=1) were collected. The type of loss incurred due to the heavy rainfall was also inquired from participants in Sample 1.

### PTSD checklist-specific stressor version

The PTSD checklist-specific stressor version (PCL-S) (Weather, Litz, Herman, Huska, & Keane, 1993) is a self-report instrument including 17 items that correspond to the 17 DSM-IV-TR (APA, 2000) symptoms of PTSD. Respondents rate each item using a five-point Likert scale (*not at all* to *extremely)*. For the current study, the respondents rated the presence of symptoms in the past month corresponding to the specific event (natural disaster or displacement). For analyses of PTSD prevalence rates, first the DSM-IV-TR symptom criteria were set including the presence of at least one item of Intrusive recollection, three items measuring Avoidant/Numbing, and at least two items from Hyperarousal, which were rated “*moderately*” *to* “*extremely*” (APA, 2000). Second, for prevalence of PTSD and for the purpose of referral, we used the overall cut-off score of 50 as recommended in previous literature (Forbes, Creamer, & Biddle, 2001). Internal consistency of the PCL was found to be 0.89 in an earlier Indian study (Suresh, Furr, & Srikrishnan, 2009). Further, PCL scores have been found to be higher in women victims of intimate partner violence from India, compared to those with no report partner violence (Chandra, Satyanarayana, & Carey, 2009). The PCL correlates moderate to high with the Clinician-Administered PTSD Scale (CAPS; Blanchard, Jones-Alexander, Buckley, & Forneris, 1996; Forbes et al., 2001). Cronbach's *α* of the PCL for the present study are presented in Table 2.

### Data analyses

Descriptive statistics and *t*-tests for comparison of the means of the two samples were conducted in IBM SPSS version 20.0 (IBM Corp., 2011). M*plus* 7.11 (Muthén & Muthén, 2013) was used to conduct several CFA, and then the MIMIC structural equation model was tested (Muthén, 1989). Based on recent literature, we tested the factor structure of PTSD according to three competing models based on DSM-IV-TR PTSD symptoms, i.e., two four-factor models namely, Emotional Numbing (King et al., 1998) and Dysphoria (Simms et al., 2002), and the five-factor model of Dysphoric Arousal (Elhai et al., 2011). Assumptions of univariate (no skewness/kurtosis values>1.35) and multivariate (Mardia's Kurtosis=45.96, *p<*0.001; DeCarlo, 1997) normality were not met. Thus, we used maximum likelihood estimation with robust standard errors (MLR) in CFA which calculates the scaled Chi-square statistic (Y–B*χ* ^2^; Yuan & Bentler, 2000), and is robust to non-normality. For all three CFAs, error covariances were fixed to zero while all the factors were inter-correlated. We used robust versions of approximate fit indices which included the comparative fit index (CFI), the Tucker–Lewis Index (TLI), the root mean square error of approximation (RMSEA), and the standardized root mean square residual (SRMR). These approximate fit indices are among the most widely used indices in structural equation modeling literature, and their use as a set provides information about the model fit (Kline, 2011). As recommended by Hu and Bentler (1999) excellent (or adequate) fit of models is considered when CFI and TLI≥0.95 (0.90–0.94), RMSEA<0.06 (to 0.08), and SRMR<0.08 (to 0.1). To compare nested models (i.e., Dysphoric Arousal vs. Emotional Numbing, and Dysphoria), we used the corrected scaled *χ* ^2^ difference test (Satorra & Bentler, 2001). Models are nested when one model (restricted model; e.g., the Emotional Numbing or Dysphoria model) is a subset of the other (full model; e.g., Dysphoric Arousal model. In other words, the full model contains all the terms of the restricted models, and an additional term. Further, comparison of the non-nested models (i.e., Emotional Numbing vs. Dysphoria) was carried out using the Bayesian information criterion (BIC; Schwarz, 1978). A BIC difference greater than 10 reflects a very strong support in favor of the model with the lower BIC value (Raftery, 1995). In a recent review, these quantitative methods have also been recommended for model comparisons of the PTSD factor structure (Elhai & Palmieri, 2011). Second, after obtaining the best fitting model, gender as a covariate was added to the CFA model (MIMIC), and the relation between gender and the factors of PTSD was assessed.

## Results

### Data screening and descriptive statistics

No missing values were found in either of the two samples. The two samples were combined for further analyses. No significant difference on mean scores on the PCL was found between the two samples (*M*_*1*_=35.44 vs. *M*_*2*_=36.59; *t* (311)=0.69, *p=*0.49). Neither the subsamples nor the total sample showed meaningful skewness or kurtosis on the total score of the PCL-S (cf. Table 2). Combination of the two samples is also justifiable given that PTSD is not trauma specific; indeed many individuals who have experienced various trauma types have experienced PTSD as a result, and the factor structure of each of the models tested herein has been shown to provide good fit to the data across a variety of trauma populations (Yufik & Simms, 2010). Among females and males, 22.3 and 18.1% met the diagnostic criteria for PTSD according to the DSM-IV-TR criteria (APA, 2000), respectively. The PTSD criterion was met by 19.4% of females and 13.8% of males who had a score of 50 or higher on PCL-S. Tables 1 and 2 present the item and score statistics, respectively.

**Table 1:** Mean, SD, skewness, and kurtosis of the DSM-IV-TR cluster items of PTSD, the three DSM-IV criteria of PTSD, and intercorrelations of the items (*N=*313)

Sx	M (SD)	Skew/Kurt	B1	B2	B3	B4	B5	C1	C2	C3	C4	C5	C6	C7	D1	D2	D3	D4	D5
B1	2.23 (1.32)	0.84/ − 0.47	1																
B2	1.71 (1.09)	1.61/1.78	0.53	1															
B3	1.98 (1.26)	1.09/ − 0.09	0.42	0.45	1														
B4	2.55 (1.44)	0.44/ − 1.19	0.48	0.47	0.46	1													
B5	1.95 (1.31)	1.13/ − 0.06	0.45	0.55	0.49	0.44	1												
C1	2.42 (1.45)	0.57/ − 1.09	0.41	0.50	0.35	0.50	0.46	1											
C2	2.18 (1.34)	0.83/ − 0.60	0.37	0.44	0.40	0.42	0.50	0.57	1										
C3	1.94 (1.24)	1.16/0.19	0.37	0.44	0.42	0.41	0.44	0.50	0.46	1									
C4	2.00 (1.27)	1.04/ − 0.15	0.30	0.27	0.32	0.36	0.36	0.25	0.38	0.19	1								
C5	1.78 (1.22)	1.48/0.99	0.34	0.30	0.45	0.36	0.42	0.35	0.42	0.37	0.46	1							
C6	1.66 (1.12)	1.73/1.98	0.28	0.26	0.38	0.22	0.43	0.29	0.43	0.38	0.40	0.59	1						
C7	2.05 (1.41)	0.98/ − 0.55	0.29	0.29	0.27	0.38	0.30	0.25	0.34	0.22	0.39	0.34	0.38	1					
D1	1.71 (1.18)	1.70/1.80	0.34	0.38	0.45	0.39	0.51	0.33	0.32	0.34	0.33	0.36	0.36	0.31	1				
D2	2.58 (1.54)	0.44/ − 1.33	0.26	0.20	0.26	0.19	0.34	0.24	0.22	0.23	0.19	0.29	0.27	0.30	0.35	1			
D3	2.17 (1.37)	0.88/ − 0.60	0.41	0.40	0.44	0.38	0.40	0.41	0.43	0.30	0.36	0.40	0.37	0.27	0.41	0.37	1		
D4	2.66 (1.46)	0.35/ − 1.25	0.43	0.34	0.41	0.42	0.41	0.43	0.37	0.31	0.36	0.42	0.30	0.28	0.36	0.22	0.39	1	
D5	2.29 (1.39)	0.73/ − 0.83	0.33	0.34	0.37	0.39	0.37	0.37	0.33	0.36	0.29	0.34	0.27	0.44	0.36	0.37	0.47	0.53	1

**Table 2:** PCL total score, PTSD means according to the three DSM-IV-TR criteria, and proportion with diagnosis of PTSD according to DSM-IV-TR and according to the PCL cutoff score

	Total (*N*=313)		Sample 1 (*N*=200)		Sample 2 (*N*=113)	
	
	Mean (SD)/percentage	Skew/Kurt	Mean(SD)/percentage	Skew/Kurt	Mean(SD)/percentage	Skew/Kurt
PCL total (*α*=**0.91**, *0.90*, 0.92a†)	35.85 (14.26)	0.75/0.00	35.44 (12.14)	0.48/ − 0.38	36.59 (17.44)	0.80/ − 0.44
Criteria B (*α*=**0.81**, *0.79*, 0.84†)	10.41 (4.89)	0.81/ − 0.07	10.41 (4.15)	0.59/ − 0.24	10.42 (6.0)	0.89/ − 0.49
Criteria C (*α*=**0.81**, *0.78*, 0.84†)	14.03 (6.18)	0.87/0.28	14.01 (5.33)	0.58/ − 0.44	14.07 (7.48)	1.0/0.13
Criteria D (*α*=**0.75**, *0.73*, 0.80†)	11.41 (4.93)	0.74/ − 0.12	11.02 (4.12)	0.59/ − 0.18	12.11 (6.07)	0.60/ − 0.83
PTSD* (DSM-IV-TR)	20.4	–	18	–	24.8	–
PTSD* (PCL cutoff≥50)	16.9	–	12	–	25.7	–

* Variable with superscript has value depicted as percentage.

† Cronbach's alpha (*α*) values in bold face are for total sample, in italics are for sample 1, and with superscript are for sample 2. Skew = skewness; Kurt = kurtosis.

### Factor structure of PTSD

Table 3 depicts the approximate fit indices for the three competing models. Noteworthy, is that all three models had adequate and similar fit. On comparing the two non-nested models, the Emotional Numbing model provided better fit compared to the Dysphoria model (ΔBIC=12.68). For nested model comparisons, the corrected scaled *χ* ^2^ difference test showed that the Dysphoric Arousal model fit significantly better than the Emotional Numbing model [▵*χ* ^2^ (4, *N=*313)=11.16, *p=*0.02], and the Dysphoria model [Δ*χ* ^2^ (4, *N=*313)=13.79, *p=*0.008]. Hence, the Dysphoric Arousal model was deemed to provide a better fit to the data compared to the other four-factor models based on the corrected scaled *χ*^2^ difference test.^1^ The standardized factor loadings and factor correlations for the five-factor Dysphoric Arousal model are presented in Table 4.

**Table 3:** Fit indices for the three models

Models	χ^2^	Y–B *χ* ^2^	*df*	CFI/TLI	SRMR	RMSEA	RMSEA 90% CI	BIC
Model 1	309.14	216.18	113^a^	0.92/0.91	0.05	0.05	0.04–0.07	16471.27
Model 2	320.67	221.15	113^a^	0.92/0.90	0.05	0.06	0.04–0.07	16483.95
Model 3	293.18	205.02	109^a^	0.93/0.91	0.05	0.05	0.04–0.06	16478.94

a Indicates significance at *p*<0.001 level. Model 1=Emotional Numbing model; Model 2=Dysphoria model; Model 3=Dysphoric Arousal model; Y–B *χ*^2^=scaled Yuan–Bentler Chi-square; CFI=comparative fit index; TLI=Tucker–Lewis index; SRMR=standardized root mean square; RMSEA=root mean square error of approximation; CI=confidence interval; BIC=Bayesian information criterion.

**Table 4:** Standardized factor loadings across the Emotional numbing model, the Dysphoria model, and the Dysphoric Arousal model, and factor correlations for the Dysphoric Arousal model

Item	Emotional Numbing		Dysphoria		Dysphoric Arousal
B1. Intrusive thoughts	0.66		0.66		0.66
B2. Nightmares	0.70		0.70		0.70
B3. Flashbacks	0.67		0.67		0.67
B4. Emotional reactivity	0.67		0.67		0.67
B5. Physical reactivity	0.74		0.74		0.74
C1. Avoidance of thoughts	0.74		0.75		0.75
C2. Avoidance of reminders	0.77		0.77		0.77
C3. Trauma-related amnesia	0.57		0.58		0.57
C4. Loss of interest	0.58		0.56		0.58
C5. Feeling detached	0.73		0.67		0.73
C6. Feeling numb	0.68		0.62		0.69
C7. Foreshortened future	0.54		0.53		0.53
D1. Sleep disturbance	0.63		0.62		0.65
D2. Irritability	0.48		0.46		0.49
D3. Difficulty concentrating	0.68		0.65		0.69
D4. Hyper-vigilance	0.65		0.73		0.73
D5. Exaggerated startle	0.66		0.72		0.72
Factor correlation for Dysphoric Arousal model	Re-experiencing	Avoidance	Numbing	Dysphoric Arousal	Anxious Arousal
Avoidance	0.83	–			
Numbing	0.79	0.76	–		
Dysphoric Arousal	0.86	0.72	0.81	–	
Anxious Arousal	0.76	0.68	0.73	0.81	–

All values are significant at *p*<0.001 level.

Next, the addition of gender as a covariate to the CFA model lead to the formation of a model with an adequate fit without alterations in the factor structure of the Dysphoric Arousal model of PTSD (Y–B *χ* ^2^ [121, *N*=313]=324.26, CFI/TLI=0.92/0.90, RMSEA=0.06 [90% CI=0.04–0.07], SRMR=0.05). Further, as shown in Table 5, gender was associated with PTSD Re-experiencing, and Anxious Arousal factors, with females reporting higher mean scores as compared to males. However, no association was found between gender and factors of Avoidance, Numbing, and Dysphoric Arousal (Table 5).

**Table 5:** Unstandardized regression coefficients of the five factors of the Dysphoric Arousal model of PTSD (DSM-IV-TR) on gender in a MIMIC model

Factors of Dysphoric Arousal model of PTSD	B	S.E.	*z*-test	Direction of effect
Re-experiencing	−0.35**	0.13	2.72	F>M
Avoidance	−0.12	0.13	0.86	–
Numbing	−0.16	0.13	1.24	–
Dysphoric Arousal	−0.14	0.15	0.95	–
Anxious Arousal	−0.44**	0.15	2.99	F>M

F>M=females have a higher score than males.

** *p*<0.01.

## Discussion

The present study aimed to assess the factor structure of PTSD symptoms as measured by DSM-IV-TR, and compared three competing models: the Emotional Numbing model, the Dysphoria model, and the Dysphoric Arousal model. Although this study was not able to directly assess the DSM-5 PTSD structure (APA, 2013), or the newly proposed six-factor models of PTSD as per DSM-5 PTSD symptoms (Liu et al., 2014; Tsai et al., in press), it tested the currently best developed empirical models of PTSD based on DSM-IV-TR criteria, presented in literature. While all three competing models had adequate and similar fit indices, our hypothesis was supported as the correlated five-factor Dysphoric Arousal model (Elhai et al., 2011) provided a better fit to the data compared to the prevailing two four-factor models (i.e., Emotional Numbing and Dysphoria) models. However, it should be noted that the current findings are valid only for the total sample and not specifically to the sub-samples. Nonetheless, the present findings are in line with recent studies from across the globe on victims of domestic violence, adolescent/adult earthquake, and riot survivors, elderly bereaved, war veterans, primary care patients, survivors of the Tsunami, World Trade Center responders, army soldiers, and epidemiological surveys (Armour et al., 2012; Armour, Carragher, et al., 2013; Armour, Ghazali, et al., 2013; Armour, O'Connor, et al., 2013; Elhai et al., 2011; Harpaz-Rotem et al., 2014; Pietrzak et al., 2014; Semage et al., 2013; Wang, Long, et al., 2011; Wang, Zhang, et al., 2011). As mentioned earlier, differences in the manifestation of symptoms of PTSD across cultures may lead to differences in the factor structure, and perhaps future revisions of PTSD criteria may see the bifurcation of the Hyperarousal factor as depicted in the Dysphoric Arousal model, which was not the case in the recently released DSM-5. However, it is important to note that the Dysphoric Arousal model provided a superior fit as compared to the Emotional Numbing model in the present study on the basis of the corrected scaled *χ* ^2^ difference test only, and hence future studies based on similar samples need to replicate the factor structure obtained herein.

The better fit of the Dysphoric Arousal model in the current study, points toward the distinctiveness of items measuring restlessness and agitation from the earlier proposed Dysphoria factor, which measures general numbing of responsiveness, and also from the remaining Hyperarousal items which are reflective of fear and panic symptoms (Watson, 2005). Additionally, the current findings also support the bifurcation of the Avoidance/Numbing factor of DSM-IV-TR, into Avoidance and NAMC (akin to numbing factor; Forbes et al., 2011), by DSM-5 (APA, 2013).

The parameter estimates of the Dysphoric Arousal model indicated that the lowest factor loading was of the item measuring irritability/anger-outbursts. This is in contrast to previous studies which often report that the item representing trauma-related amnesia provides the lowest factor loading (Elhai et al., 2011; Wang, Zhang, et al., 2011). Notably, the DSM-IV-TR PTSD symptom of irritability/anger outburst has been refined in the DSM-5 (APA, 2013), as it conflated an emotional state with a behavioral action (Friedman, 2013). In the current version of the DSM, the refined item adhering to feeling anger is included alongside other negative emotions (Criteria D), whereas the behavioral symptom is under Alternations in arousal and reactivity (Criteria E). Additionally, Tsai et al. (in press) in their recently proposed six-factor PTSD model based on the DSM-5 PTSD symptoms, built on the Dysphoric Arousal model and added a sixth factor of “externalizing behavior.” This cluster includes the two items of irritability/anger and reckless behavior. However, assessing the same is beyond the scope of the present study, and future factor analytic studies should test for the six-factor models of PTSD. Furthermore, in the present study, all inter-factor correlations were found to be moderate to high (0.68–0.86). The factor correlations between Dysphoric Arousal and Numbing, and between Dysphoric Arousal and Anxious Arousal in the current study may generally be regarded as high (0.81, cf. Table 3). However, as these factors represent an overarching construct of PTSD high inter-factor correlations are to be expected (Wang, Zhang, et al., 2011). Nonetheless, external measures of psychopathology in the form of clinical diagnosis and assessment are needed to emphasize the distinction between the factors, and the present study is limited in this regard.

The present findings further indicate that females and males differ on the factor means of Re-experiencing and Anxious Arousal, with females scoring higher. However, no gender difference in mean scores was found on the remaining three factors. Therefore, our hypothesis that females and males would differ in level of symptoms across all the factors of PTSD was only partially supported. It is important to mention that the females in the present study had overall higher PTSD scores compared to males; a finding in line with other studies from India and the West (John et al., 2007; Kessler et al., 1995; Kumar et al., 2007). However, the empirically documented two-fold increase in PTSD in females as compared to males (Kessler et al., 1995; Tolin & Foa, 2006), was not evident in the present study. Notably, related studies from India, fail to show the likelihood of females experiencing PTSD twice as much as males (Contractor et al., 2014; Kar et al., 2007). However, these studies as well as the present study did not assess for lifetime PTSD, and focused on analysis of PTSD based on one specific event (e.g., terrorist attack, natural disaster). Future studies may want to consider various lifetime events (e.g., sexual assault, natural disaster) that can lead to PTSD. Furthermore, the higher factor mean scores on Re-experiencing and Anxious Arousal for females appear to be in line with literature reporting more physiological reaction among females than males when faced with trauma stimuli (Shalev, Orr, & Pitman, 1993). However, the absence of association between gender and the factors of Avoidance, Numbing, and Dysphoric Arousal stands in contrast to more recent literature conducted on samples from China (Wang et al., 2013) and the US (Contractor et al., 2013) employing the Dysphoric Arousal model of PTSD. Notably, these studies used an adolescent sample. A potential reason for the observed gender differences that warrants future research, may be the coping style employed by females and males in the present study. A tendency to cope at a societal level when faced with a trauma incident experienced by all (Rajkumar et al., 2008) may have led to overcoming traditional gender-role expectations leading to expression of distress in males or use of problem-focused coping by females. This may have led to the lack of differences across gender in some factors of PTSD.

The present study should be interpreted with the following limitations in mind. First, the exclusive reliance on self-report for assessing PTSD is a methodological limitation. Furthermore, we used a single measurement tool to assess PTSD and its latent structure. We must be mindful that by doing so we are assessing the tool itself as a proxy of PTSD. Second, the PCL was not back-translated as the two languages employed during the administration of the tools were neither the national language (i.e., Hindi) nor the official state language (i.e., Urdu). The present study is limited in this regard. Future studies should focus on translations and back-translations of the PCL into regional languages, and also validating the PCL against tools such as the CAPS (Blanchard et al., 1996). However, studies such as the present one, attempt to fill the gap between research from high income nations from the west which dominate the traumatic stress literature, and the lack of research from the low- and middle-income countries like India (Fodor et al., 2014). Third, the present study is limited in its inability to extract and separate the various kinds of traumatic experiences from the specified trauma events.

Notwithstanding these limitations we conclude that the present study, utilizing a novel, non-western sample, contributes to the existing debate on the underlying dimensionality of PTSD in favor of the Dysphoric Arousal model based on DSM-IV-TR PTSD symptoms, and its applicability across nations. The inclusion of gender as a covariate further highlights the difference between females and males on the factors of the Dysphoric Arousal model, indicating that females score higher on certain factors which are most related to physiological reactions. Surpassing the social milieu these findings have clinical implications and emphasize that while females are physiologically more reactive than males to trauma stimuli (Norris et al., 2001), male reports on other factors of PTSD are similar to those of females.

## Supplementary Material

Factor structure of PTSD, and relation with gender in trauma survivors from India

Factor structure of PTSD, and relation with gender in trauma survivors from India

Factor structure of PTSD, and relation with gender in trauma survivors from India

## Footnotes

1 As recommended, using confirmatory factor analyses, the three competing PTSD models were tested across the two samples separately. Noteworthy is that in sample 1 (*N*=200), the fit indices for all the three models were adequate. Further, model comparison did not reveal any one model to be better than the other. For sample 2 (*N*=113), the fit indices were overall weak, and hence no model comparisons were conducted.
