# Supplementary material for: Factor structure of PTSD, and relation with gender in trauma survivors from India
Source: Eur J Psychotraumatol. 2014 Nov 17;5:10.3402/ejpt.v5.25547. doi: 10.3402/ejpt.v5.25547 (PMC4247496; doi:10.3402/ejpt.v5.25547)
Supplement: Factor structure of PTSD, and relation with gender in trauma survivors from India [file EJPT-5-25547-s001.pdf]

TSSB'nin Faktör yapısı ve Hindistan'daki travma yaşamış kişilerde cinsiyetle ilişkisi

Ruby Charak, Cherie Armour, Ask Elklit, Disket Angmo, Jon D Elhai, Hans M Koot

Arkaplan: Batı kültürlerinde TSSB'nin faktör yapısı kapsamlı bir şekilde araştırılmıştır. Bazı çalışmalar bu faktör yapısını Asya'da incelemiştir (Çin, Sri Lanka ve Malezya); ancak çok az araştırma TSSB'nin faktör yapısını yetişkin Hintli örneklemlerde doğrudan incelemiştir. Ayrıca, güçlü cinsiyet rollerinin var olduğu büyük ölçüde ataerkil bir toplum olan Hindistan'da cinsiyet ve TSSB faktörleri arasındaki ilişkinin incelenmesi zaruridir.
Amaç: Mevcut araştırmanın amacı DSM-IV-TR (APA, 2000)'de tanımlanan TSSB'nin geçerli modellerini temel alarak travma yaşamış Hintli bir örneklem üzerinde TSSB faktör yapısını incelemektir.
Yöntem: Örneklem, Hindistan'ın Cemmu ve Keşmir eyaletindeki doğal afet yaşamış (N=200) veya sınır ötesi silahlı çatışmalar sebebiyle yer değiştirme (N=113) yaşamış olan 313 katılımcıdan (% 55.9 kadın) oluşmaktadır.
Sonuçlar: Var olan üç TSSB modeli - iki tane dört faktörlü model (Duygusal Hissizleşme ve Disfori) ve bir tane beş faktörlü model (Disforik Uyarılma) - cinsiyet eşdeğişken olarak eklenerek doğrulayıcı faktör analizi ile test edilmiştir. Disforik uyarılma modeli Duygusal Hissizleşme ve Disforik modellere kıyasla anlamlı olarak daha fazla uygunluk gösterse de bu üç model birbirine benzer uyum indeksleri göstermiştir. Cinsiyet farkları tekrar deneyimlime ve kaygılı uyarılma faktörleri üzerinde farklılık göstermiştir.
Tartışma: Uyum indeksleri açısından bütün modeller oldukça benzerlik gösterse de bulgular TSSB'nin Disforik Uyarılma modelinin en iyi model olduğunu göstermektedir. Erkeklere kıyasla kadınlar tekrar deneyimleme ve kaygılı uyarılma faktörlerinde daha yüksek skorlar almışlardır. TSSB'nin iki faktörü üzerinde görülen cinsiyet farkları Hindistan'daki sosyal çevre ışığında tartışılmıştır.

Anahtar kelimeler: TSSB; faktör yapısı; cinsiyet; disforik uyarılma modeli; MIMIC; Doğal afet; savaş sonucu yer değiştirme.

Name of translator: Emek Yuce Zeyrek-Rios

Citation: European Journal of Psychotraumatology 2014, 5: 25547 - http://dx.doi.org/10.3402/ejpt.v5.25547
