# Supplementary material for: Factor structure of PTSD, and relation with gender in trauma survivors from India
Source: Eur J Psychotraumatol. 2014 Nov 17;5:10.3402/ejpt.v5.25547. doi: 10.3402/ejpt.v5.25547 (PMC4247496; doi:10.3402/ejpt.v5.25547)
Supplement: Factor structure of PTSD, and relation with gender in trauma survivors from India [file EJPT-5-25547-s002.pdf]

Structure factorielle de l'état de stress post-traumatique et relation avec le sexe chez des survivants de traumatismes en Inde

Ruby Charak, Cherie Armour, Ask Elklit, Disket Angmo, Jon D Elhai, Hans M Koot

Contexte : la structure factorielle de l'état de stress post-traumatique (ESPT) a été amplement étudiée dans les pays occidentaux. Certaines études ont évalué cette structure factorielle en Asie (Chine, Sri Lanka et Malaisie), mais peu ont directement évalué la structure factorielle de l'ESPT dans un échantillon d'indiens adultes. De plus et dans une société largement patriarcale avec des rôles forts en fonction du sexe de l'individu, il devient impératif d'évaluer l'association entre les facteurs d'ESPT et le sexe.
Objectif : le but de la présente étude a été 1) d'évaluer la structure factorielle de l'ESPT dans un échantillon indien de survivants à un traumatisme, basée sur les modèles d'ESPT dominants comme définis dans le DSM-IV-TR (APA, 2000), et 2) d'évaluer la relation entre les facteurs d'ESPT et le sexe.
Méthode : l'échantillon comprenait 313 participants (55,9% femmes) provenant de Jammu et Kashmir (Inde) et ayant fait l'expérience d'une catastrophe naturelle (N = 200) ou de relocalisations dues à des conflits frontaliers (N = 113).
Résultats : trois modèles d'ESPT existants – deux modèles à 4 facteurs (engourdissement émotionnel et dysphorie), et un modèle à 5 facteurs (excitation dysphorique, *ndt. dysphoric arousal*) – ont été évalués par l'Analyse de Facteur Confirmant (AFC) avec ajout du sexe comme variable indépendante. Ces trois modèles en compétition avaient des indices de correspondance similaires bien que le modèle d'excitation dysphorique correspondait significativement mieux que les modèles d'engourdissement émotionnel et de dysphorie. Des différences entre genres ont été trouvées sur les facteurs de reviviscence et d'excitation anxieuse (*ndt. anxious arousal*).
Conclusions : les résultats indiquent que le modèle d'excitation dysphorique de l'ESPT est le meilleur modèle, bien que les indices de correspondance de tous les modèles aient été similaires. Comparés aux hommes, les femmes avaient des scores plus élevés sur les facteurs de reviviscence et d'excitation anxieuse. Les différences entre sexes, trouvées sur ces deux facteurs d'ESPT, sont discutées dans le contexte social de l'Inde.

Keywords: ESPT, structure factorielle, sexe, modèle d'excitation dysphorique, MIMIC, catastrophe naturel, relocalisation liée à la guerre

Name of translator: Marc Legrand

Citation: European Journal of Psychotraumatology 2014, 5: 25547 - http://dx.doi.org/10.3402/ejpt.v5.25547
