# Supplementary material for: Factor structure of PTSD, and relation with gender in trauma survivors from India
Source: Eur J Psychotraumatol. 2014 Nov 17;5:10.3402/ejpt.v5.25547. doi: 10.3402/ejpt.v5.25547 (PMC4247496; doi:10.3402/ejpt.v5.25547)
Supplement: Factor structure of PTSD, and relation with gender in trauma survivors from India [file EJPT-5-25547-s003.pdf]

Факторный состав ПТСР и его связь с гендером у пострадавших от травм в Индии

Ruby Charak, Cherie Armour, Ask Elklit, Disket Angmo, Jon D Elhai, Hans M Koot

Аннотация
История вопроса: Факторный состав ПТСР был тщательно изучен в западных странах. Некоторые исследования оценили факторную структуру ПТСР в Азии (Китай, Шри-Ланка и Малайзия), но мало кто напрямую изучал ее на примере взрослых жителей Индии. К тому же, учитывая значительную степень патриархальности общества в Индии с ярко выраженными гендерными ролями, актуальной задачей становится оценка связи между факторами ПТСР и гендером.
Цель: Целью данного исследования была оценка факторной структуры ПТСР на примере пострадавших от травмы индийцев, основанная на определенных в DSM-IV-TR (APA, 2000) моделях ПТСР, а также оценка связи между факторами ПТСР и гендером.
Метод: Выборка состояла из 313 участников (55,9% женщин) из индийского штата Джамму и Кашмир, которые пережили стихийное бедствие (N = 200) или смену места жительства из-за трансграничных конфликтов (N = 113).
Результаты: Три существующие ПТСР модели – две четырехфакторные (Эмоциональное онемение и Дисфория) и одна пятифакторная (Дисфорическое возбуждение) – были протестированы с использованием подтверждающего факторного анализа (CFA) с добавлением гендера в качестве коварианты. Три конкурирующие модели имели подобные подходящие индексы, хотя модель Дисфорическое возбуждение подходит значительно лучше, чем модели Эмоциональное онемение и Дисфория. Гендерные различия были обнаружены по факторам Преодоление и Тревожное возбуждение.
Выводы: Полученные результаты свидетельствуют о том, что модель ПТСР Дисфорическое возбуждение оказалась лучшей моделью; хотя соответствующие индексы всех моделей были довольно похожи. По сравнению с мужчинами, женщины набрали больше баллов по факторам Преодоление и Тревожное возбуждение. Гендерные различия, найденные в двух факторах ПТСР, объясняются особенностями социальной среды в Индии.

Ключевые слова: ПТСР, факторная структура, гендер, модель Дисфорическое возбуждение, MIMIC, стихийное бедствие, беженцы

Name of translator: Marina Shcherbak

Citation: European Journal of Psychotraumatology 2014, 5: 25547 - http://dx.doi.org/10.3402/ejpt.v5.25547
